# Isolation, identification, and characterization of corn-derived antioxidant peptides from corn fermented milk by *Limosilactobacillus fermentum*

**DOI:** 10.3389/fnut.2022.1041655

**Published:** 2022-11-09

**Authors:** Jue Xu, Yingyan Chen, Xiankang Fan, Zihang Shi, Mingzhen Liu, Xiaoqun Zeng, Zhen Wu, Daodong Pan

**Affiliations:** ^1^Key Laboratory of Animal Protein Food Deep Processing Technology of Zhejiang Province, College of Food and Pharmaceutical Sciences, Ningbo University, Ningbo, China; ^2^State Key Laboratory for Managing Biotic and Chemical Threats to the Quality and Safety of Agro-products, Ningbo University, Ningbo, China; ^3^Department of Food Science and Technology, School of Food Science and Pharmaceutical Engineering, Nanjing Normal University, Nanjing, China

**Keywords:** lactic acid bacteria, antioxidant peptide, fermented milk, corn (maize), LC-MS/MS

## Abstract

Dairy-derived peptides and corn-derived peptides have been identified as essential ingredients for health promotion in the food industry. The hydrolysis based on lactic acid bacteria (LAB) protease system is one of the most popular methods to prepare bioactive peptides. The objectives of this paper are to develop antioxidant fermented milk and to obtain natural antioxidant peptides. In our study, LAB with antioxidant capacity were screened *in vitro*, and the corn fermented milk with antioxidant capacity was achieved by the traditional fermentation method. Fermented milk was purified by ultrafiltration and molecular sieve, and identified by liquid chromatography-tandem mass spectrometry (LC-MS/MS). Our findings demonstrate that *Limosilactobacillus fermentum* L15 had a scavenging capacity of more than 80% of DPPH radicals, Trolox equivalent antioxidant capacity (TEAC) of 0.348 ± 0.005 mmol/L. Meanwhile, the peptide content of corn fermented milk prepared with *L. fermentum* L15 was 0.914 ± 0.009 mg/mL and TAEC of 0.781 ± 0.020 mmol/L. Particularly important, IGGIGTVPVGR and LTTVTPGSR isolated and extracted from fermented milk were found to have antioxidant capacity for the first time. The synthetic peptides IGGIGTVPVGR and LTTVTPGSR demonstrated a scavenging capacity of 70.07 ± 2.71% and 70.07 ± 2.77% for DPPH radicals and an antioxidant capacity of 0.62 ± 0.01 mmol/L and 0.64 ± 0.02 mmol/L Trolox equivalent, respectively. This research provides ideas and basis for the development and utilization of functional dairy products.

## Introduction

Oxidation is an inevitable process in the oxidative metabolism of all organisms (1). However, the imbalance between oxidation and antioxidation causes oxidative stress, which is one of the major causes of various chronic diseases such as aging, cardiovascular diseases, diabetes, Alzheimer's disease, and cancer (2, 3). Synthetic antioxidants, although having strong antioxidant activity, are potentially risky in the organism, so attention has been turned to natural antioxidants, and many studies revealed that peptides produced by the hydrolysis of various proteins or by microbial fermentation have antioxidant activity (4). Therefore, the development of antioxidant products from natural foods has become a current research hotspot for antioxidants. Fermented dairy products are becoming increasingly popular with consumers, especially those who have special nutritional requirements. In addition to the rich nutrients in milk, the bioactive peptides produced by lactic acid bacteria (LAB) breaking down milk proteins are also an excellent resource. Fermentation is an effective way to produce target bioactive peptides at this stage, and numerous studies have demonstrated the production of bioactive peptides by LAB through a protein hydrolysis system, and these active peptides have anticoagulant, angiotensin-converting enzyme activity inhibition and antioxidant capacity (5). These natural exogenous antioxidants have received attention from the food industry and the medical field, and the fact that they can be supplemented without limit and do not cause dangerous immune reactions in the body provides an effective way to alleviate oxidation.

In fermented milk, the content of antioxidant peptides was dependent on the type of probiotics used. It is well-known that probiotics contain a lot of enzymes. LAB is the most widely used probiotic microorganisms. The proteins in milk were degraded into different polypeptides by the cell envelope protease (CEP) on the surface of LAB during fermentation process (6). Degradation products are transported across the cell membrane by different peptide transport systems, including oligopeptide (Opp), dipeptide (DtpP), and tripeptide (DtpT) transport carriers (7). They are then hydrolyzed into amino acids or small peptides by intracellular peptidase, and produce a series of bioactive peptides. Our previous study demonstrated that fermented milk of *Lactobacillus delbrueckii* subsp. *bulgaricus* has an antioxidant capacity and the hydrolysis of casein and whey proteins by *Lactobacillus reuteri* released bioactive peptides with more potential antioxidant capacity compared to *Lactobacillus brevis* and *Lactobacillus plantarum* (8). Other studies have also illustrated that the fermentation of milk by *Lactobacillus acidophilus* increases the production of bioactive peptides, especially antioxidant peptides (9). The peptides extracted from *kefir*, a fermenting agent rich in probiotics (LAB, acetate bacteria, and yeast), had also shown potent and excellent functional properties (10). Screening of LAB that produce antioxidant peptides is essential to obtain bioactive peptides in fermentation.

Plant foods not only offer many potential health benefits, but they are also rich in protein. Plant ingredients are typically added to foods to increase nutritional value (11). Corn is one of the most important foods and industrial crops in the world, and has a comprehensive nutritional profile. Corn contains active oxidizing substances such as phenolic acids, anthocyanins, carotenoids, etc. (12). The antioxidant capacity of corn had been well-known. Corn silk polysaccharide had a good antioxidant capacity and protects oxidatively damaged renal epithelial cells (13). Zeaxanthin and lutein in corn had also been reported to have strong antioxidant activity, significantly reducing visual fatigue and reducing the risk of macular degeneration and cataracts (14). Corn also contains 10–15% protein. Corn has a protein content of 60–70% after crushing. The relatively low content of certain essential amino acids such as lysine and tryptophan results in poor-quality corn protein lacking desirable functional properties (15). Hence it is crucial to hydrolyze corn protein to improve protein utilization. Hu et al. (16) reported the enhancement of corn protein amount using hydrolyzed corn protein powder such as papain. Jorge et al. (17) measured the antioxidant capacity of corn alcohol-soluble protein on hepatocytes. Zhuang et al. (18) obtained antioxidant peptides by hydrolyzing corn protein powder with alkaline protease and flavored protease, which improved the deficiency of poor protein quantity of corn. Jang et al. (19) improved the nutrition, antioxidant, and bioavailability of corn bran and wheat bran mix by fermenting them with LAB and acid protease. However, it is rare to find studies on the fermentation of corn proteins in combination with milk proteins by LAB.

Due to the outstanding antioxidant potential of milk and corn proteins and the limited information on the production of bioactive peptides from milk fermented with *Limosilactobacillus fermentum*. This study expected to develop a corn fermented milk with the hope that its antioxidant capacity would fill the gap in developing a healthy antioxidant product from natural foods. Furthermore, the potential antioxidant peptides in the fermented milk were identified by ultrafiltration purification and LC-MS/MS. Finally, the structure-activity characteristics of the peptides were analyzed and validated.

## Materials and methods

### Screening of antioxidant peptides production capacity of LAB from fermented milk

#### Fermented milk preparation and peptide content assay

The 20 strains of LAB preserved in our laboratory were activated. The activated bacteria were inoculated into sterile skim milk at an inoculum level of 5.0% (v/v), mixed thoroughly, and then placed in a constant temperature incubator at 42°C to ferment until all the milk was curdled, and stored at 4°C for subsequent experiments.

Glutathione was used as the standard, and the peptide content was determined by the precipitation of macromolecular protein with trichloroacetic acid (TCA) and the biuret method (20). Concretely, glutathione was prepared as standard solutions with different concentrations (0, 0.4, 0.6, 0.8, 1, 1.2 mg/mL), and the absorbance values at 540 nm were measured by the biuret method and the standard curves were plotted. The peptide content of each fermented milk sample was calculated by precipitating the macromolecular proteins in the fermented milk samples with 10% trichloroacetic acid, centrifuging (4 × 103 *g*, 20 min) the supernatant, and adding bicarbonate reagent to determine the absorbance value at 540 nm.

#### Total acid and free acid in fermented milk assay

The titration acidity was determined in accordance with the National Standard of the People's Republic of China (GB 5413.34-2010) (21). Briefly, 10 *g* of fermented milk sample was mixed with 20 mL of boiled deionized water and titrated with 0.1 mol/L NaOH and phenolphthalein as an indicator. Titratable acidity was expressed as milliliters of NaOH (0.1 mol/L) consumed for the acidity value of fermented milk. The pH of the fermented milk sample was measured by recording by pH meter (Shanghai Yimai Instrument Technology Co., Shanghai, China).

#### Water-holding capacity and antioxidant activity of fermented milk

Based on the measurements by Sodini et al. (22), the WHC of the sample was measured. A homogenized sample of fermented milk (Y) was centrifuged at 4 × 10^3^ *g* for 10 min at 4°C, and the whey supernatant, removed from the sample, was weighed (WE). The WHC is calculated as follows:


$$\begin{array}{l} WHC\;\left(\%\right)=\frac{Y-WE}{Y}\times \;100\% \end{array}$$

The antioxidant capacity of LAB fermented milk was determined by the Trolox equivalent antioxidant capacity assay using the colorimetric Total Antioxidant Capacity Assay kit (Nanjing Jiancheng Bioengineering Institute, Nanjing, China). Results were obtained by interpolation to a Trolox (reference antioxidant) standard curve and expressed as Trolox equivalent antioxidant capacity (TEAC) (23). In addition, 2,2-Diphenyl-1-picrylhydrazyl (DPPH) radical scavenging activity was chosen to assess the antioxidant capacity of fermented milk (24).

#### LAB cell-envelope-proteinase assay

Lactobacillus can use cell-envelope-proteinase (CEP) to hydrolyze milk proteins into a series of short peptides, which is important for screening antioxidant peptide-producing LAB. We modified the CEP assay method, based on Sinsuwan et al. (25). After lysis of the bacterium using lysozyme, protease activity was measured at an absorbance of 410 nm using MeOsuc-Arg-Pro-Tyr-pNA (MS-Arg) as a specific substrate. Enzyme activity was defined as an increase of 0.10 per hour at 410 nm at 37°C indicating one unit of activity.

### Identification of target LAB

LAB with superior fermentability and high production of antioxidant peptides were further identified by 16S rRNA analysis. The 16S rRNA sequence analysis procedure was as follows: total DNA of a strain was extracted using a bacterial genomic DNA extraction kit. The general primers 1492R and 27F were used for amplification. The PCR fragments (1,500 bp) were purified using a quick PCR purification kit and sequenced by Sangon Biotech (Shanghai, China). Then, a phylogenetic tree was constructed by generating a complete alignment of the 16S rRNA gene of the selected members in GenBank by using MEGA software (https://www.megasoftware.net/) bootstrap values. The identified strains were finally selected for subsequent testing.

### Fermented milk with corn preparation

Alkaline protease was added to the milled corn to prepare corn digest A. Add 6% (v/v) Bacillus subtilis to A, ferment at 37°C for 18 h to fully hydrolyze the corn protein, and sterilize to prepare fermentation broth B. In the milk fermentation process, 9% (v/v) corn fermentation solution B was added to fresh milk and sterilized at 121°C for 5 min, then yogurt fermenter *Lactobacillus bulgaricus* and *Streptococcus thermophilus* (3% (v/v) inoculum), and *Limosilactobacillus fermentum* L15 (2% (v/v) inoculum) were added to make corn fermented milk at 42°C (26).

### Purification of antioxidant peptides

#### Preparation of whey

The pH of the fermented milk was adjusted to 3.4–3.6 with 1 mol/L HCl, centrifuged at 8 × 10^3^ *g* for 15 min, and the supernatant was taken, then the pH of the fermented milk was adjusted to 8.3 with 1 mol/L NaOH, centrifuged at 8 × 10^3^ *g* for 15 min, and the supernatant was collected for the subsequent extraction of peptides.

#### Ultrafiltration

Protein was concentrated by centrifugal ultrafiltration. The above samples were purified using centrifugal ultrafiltration with 3 and 10 kDa molecular weight cutoff filters to obtain fractions of different molecular weights and freeze-dried them (27). The antioxidant capacity of freeze-dried samples was assessed by two well-established methods, including DPPH radical scavenging activity and TEAC (28). The fraction with the highest antioxidant activity was selected for the next step of isolation and purification.

#### Sephadex G-25 gel filtration

A gel chromatographic column filled with Sephadex G25 was used to separate the peptides. The chromatographic column was equilibrated and the filtered sample was loaded at a flow rate of 1 mL/min. The fractions with absorption peaks at 280 nm were collected and lyophilized (29). The lyophilized peptides were dissolved in water and assayed for their antioxidant activity. The fraction with the highest antioxidant activity was selected for subsequent analysis.

### Identification of antioxidant peptides by LC-MS/MS and evaluation of antioxidant activity

To determine the peptide sequences, the Q Exactive Plus liquid mass spectrometry system (Thermo Fisher Scientific, Waltham, MA, USA) was used for analysis. Briefly, peptide samples were aspirated by the autosampler and bound to a C18 capture column (3 μm 120 Å, 100 μm 20 mm) and eluted to an analytical column (2 μm, 120 Å, 750 μm × 150 mm) for separation. Analytical gradients were established at a flow rate of 300 μL/min using two mobile phases (mobile phase A: 3% dimethyl sulphoxide (DMSO), 0.1% formic acid, 97% H_2_O, and mobile phase B: 3% DMSO, 0.1% formic acid, 97% ACN). The most abundant precursor ions were dynamically selected from a survey scan (350–1,800 m/z) of HCD fragments during mass spectrometry DDA mode analysis. The automatic gain control (AGC) target was set to 3 × 10^6^ with a maximum injection time of 20 ms and a dynamic exclusion time of 35 s. Survey scans were performed at a resolution of 70,000 at m/z 200, HCD spectral resolution was set to 17,500 at m/z 200, normalized collision energy was 28 eV, and the screening window of the four-stage rod was set to 1.6 Da. The instrument was run with the peptide recognition mode enabled.

The mass spectrometry data generated by Q Exactive Plus is retrieved through ProteinPilot (V4.5) using the database retrieval algorithm Paragon. Search the proteome reference database using Maize_Bovine_Lab in UniProt. Search parameters were as follows: Sample Type selected Identification; Cys Alkylation selected Iodoacetamide; Digestion selected Trypsin; Search Effort set to Rapid ID. Search results were filtered by Unused ≥ 1.3, entries retrieved from the inverse library and contaminating proteins were deleted, and the remaining identification information was used for subsequent analysis. The identified peptides were sent to Shanghai Botai Biotechnology Co., Ltd. for synthesis according to their sequences. The synthesized peptides were verified by HPLC-MS to determine their molecular weight. The antioxidant capacity of the synthetic peptides was assessed by the DPPH radical scavenging activity and TEAC.

### *In silico* analysis

ChemDraw online tools were used to map peptide chemical structures. The iso-electric point (pI) was analyzed by the online Innovagen server, available at www.innovagen.com/proteomics-tools. The PepDraw server (http://www.tulane.edu/~biochem/WW/PepDraw/) was used to evaluate the hydrophobicity and net charge (at neutral pH) of the peptides.

### Statistical analysis

All experiments were repeated 3 times. All statistical analyses were performed by GraphPad Prism 7.0 software. Data were expressed as mean ± SD and analyzed by repeated measures with the one-way analysis of variance (ANOVA). Bonferroni's test group comparisons were also adopted in the group data analysis. *p* < 0.05 was considered to be statistically significant, and *p* < 0.01 was considered extremely significant.

## Results and discussion

### Quality analysis of fermentation strains

**Figure 1:**
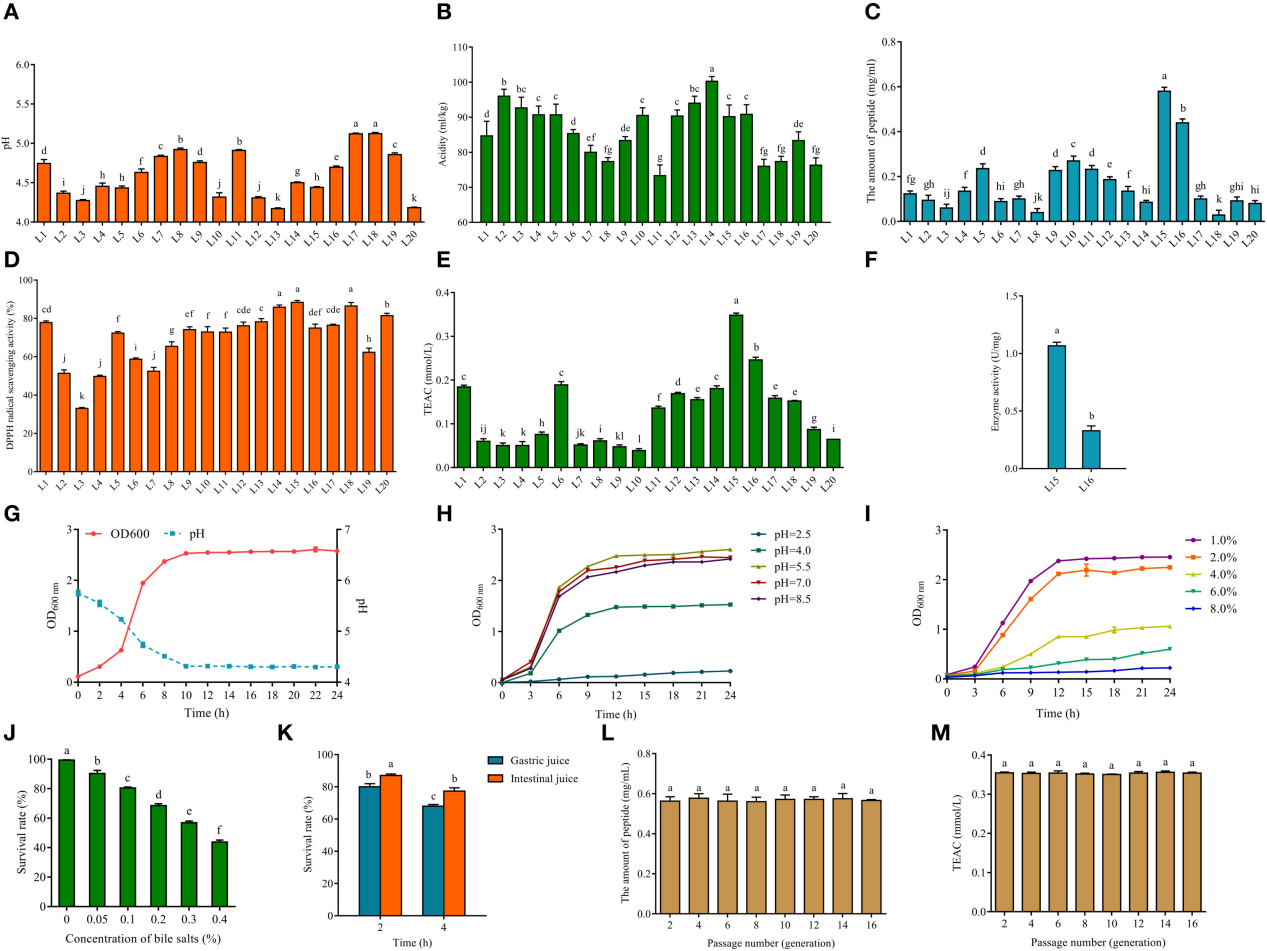
pH **(A)** and acidity **(B)** of fermented milk produced from screened LAB (L1-20). The amount of peptide **(C)** of fermented milk of 20 screened LAB (L1-20). The DPPH radical scavenging ability **(D)** and Trolox equivalent antioxidant capacity (TEAC) **(E)** of fermented milk of 20 screened LAB (L1-20). Enzyme activity **(F)** of fermented milk of L15 and L16. Growth curve and acid production capacity of *L. fermentum* L15 **(G)**. **(H)** Growth characteristics (optical density, OD_600nm_) of *L. fermentum* L15 grown in MRS medium at different pH values (F; 2.5, 4.0. 5.5, 7.0, and 8.5). **(I)** Growth characteristics (optical density, OD_600nm_) of *L. fermentum* L15 grown in MRS medium at different NaCl concentrations (w/v; 1, 2, 4, 6, and 8%). **(J)** The survival rate of *L. fermentum* L15 treated for different concentrations of bile salts. **(K)** The simulated gastrointestinal fluid environment *in vitro* on *L. fermentum* L15 growth. Genetic stability of peptide production **(L)** and TEAC **(M)** of fermented milk of *L. fermentum* L15. Different lowercase letters indicate significant differences between groups (*p* < 0.05).

In order to assess the quality of the fermented strains, this study examined the fermentation characteristics of fermented milk samples in terms of both pH and titratable acidity. The pH value of fermented milk during its shelf life is generally 4.0–4.6, and the titratable acidity is generally between 70 and 110 mL NaOH/kg (30). A higher pH value indicates inappropriate fermentation and does not effectively inhibit the growth of negative microorganisms. Islam et al. (31) examined different varieties of fermented milk marketed and found a significant difference (*p* < 0.05) in the pH of fermented milk, the average pH of the fermented milk samples remained slightly acidic, averaging between 5.28 and 6.33. In this study, there were 10 strains of milk fermented by bacteria with pH values in the range of 4.0–4.6, but all the pH values of fermented milk were below 5.5, which may be related to single bacteria fermentation (Figure 1A). Total titratable acidity is the total amount of hydrogen ions in the fermented milk sample, except for binding to basic ions (32). Therefore, titratable acidity measurements are more relevant in the evaluation of microbial fermentation capacity. Figure 1B indicates that the acidity values of each fermented buttermilk ranged from 70 to 110 mL NaOH/kg. These data show that all strains of fermented milk were of good quality and could be used for subsequent experiments.

### Isolation, identification, and preservation of antioxidant peptides producing LAB from fermented milk

The antioxidant peptide is a bioactive peptide that can maintain the balance of free radicals and enhance the anti-aging ability of the body (33). Milk contains rich protein and is one of the main sources of bioactive peptides. LAB can produce antioxidant peptides using milk proteins. Thus, it is crucial to find excellent strains for preparing antioxidant peptides. Research has demonstrated that proteolytic strains contribute to the availability of free amino acids and peptides in fermented milk (34). The main production methods of bioactive peptides are protein enzymatic digestion and dairy product fermentation (35). Thus, 20 strains of LAB were selected to ferment milk to produce peptides and to determine the peptide content and their antioxidant capacity, respectively, in this study. Figure 1C clearly showed that L15 produced the highest amount of peptides (close to 0.6 mg/mL) from fermented milk and L16 produced more than 0.4 mg/mL of peptides. The fermented milk of strain L15 showed the strongest capability of scavenging DPPH radicals (Figure 1D), and all strains except strain L3 showed excellent scavenging capability of DPPH radicals. TEAC experiments further confirmed that strain L15 fermented milk exhibited the strongest antioxidant activity among all 20 isolates (Figure 1E). The screening of *Lactobacillus delbrueckii* subsp. *bulgaricus* L7 from Xinjiang cheese in our previous study showed a good antioxidant capacity. The DPPH radical scavenging capacity of L7 fermented milk was around 80% and the TEAC was around 0.15 mmol/L. In this study, strain L15 fermented milk had a DPPH radical scavenging capacity of >80% and TEAC of 0.348 ± 0.005 mmol/L, which is better than our previous study (36).

Besides, Cell-Envelope-Proteinase (CEP) is an enzyme in the protein system of LAB, which is a key enzyme for the hydrolysis of proteins into oligopeptides and then translocation into the cell. It is also an essential component for the utilization of bovine milk proteins by LAB (37). Figure 1F reveals that the CEP enzyme activity of L15 is more than two times higher than that of L16. Therefore, L15 was selected for subsequent physiological characterization of the strain in this study (Figures 1G–K). Figure 1G showed that strain L15 reached the logarithmic stage in 2 h and reached its lowest value in 10 h, which was beneficial for fermented milk fermentation. To function in the acidic environment of the stomach, LAB must have a considerable acid tolerance (38). It was evident from Figure 1H that strain L15 was still in a growth condition even if growth of strain L15 was hindered at pH 2.5 as compared to pH 4.0. This was superior to the acid tolerance of *Lactiplantibacillus plantarum* subsp. *plantarum* F8 that we previously reported (39). Strain L15 maintained a growth trend at NaCl concentrations up to 6% (Figure 1I) and had a survival rate >40% at 0.4% bile salt (Figure 1J). This strain showed better osmotic stress tolerance and bile salt tolerance than we previously screened *Levilactobacillus brevis* 54 (26). Besides, the survival rate of L15 was more than 60% after 4 h of gastrointestinal fluid stress (Figure 1K). Moreover, the antioxidant stability of L15 was found to remain consistent over 16 h of growth in this study (Figures 1L,M, *p* > 0.05). Taking into account the antioxidant capacity and fermentation ability of the strain, strain L15 was applied in subsequent studies.

**Figure 2:**
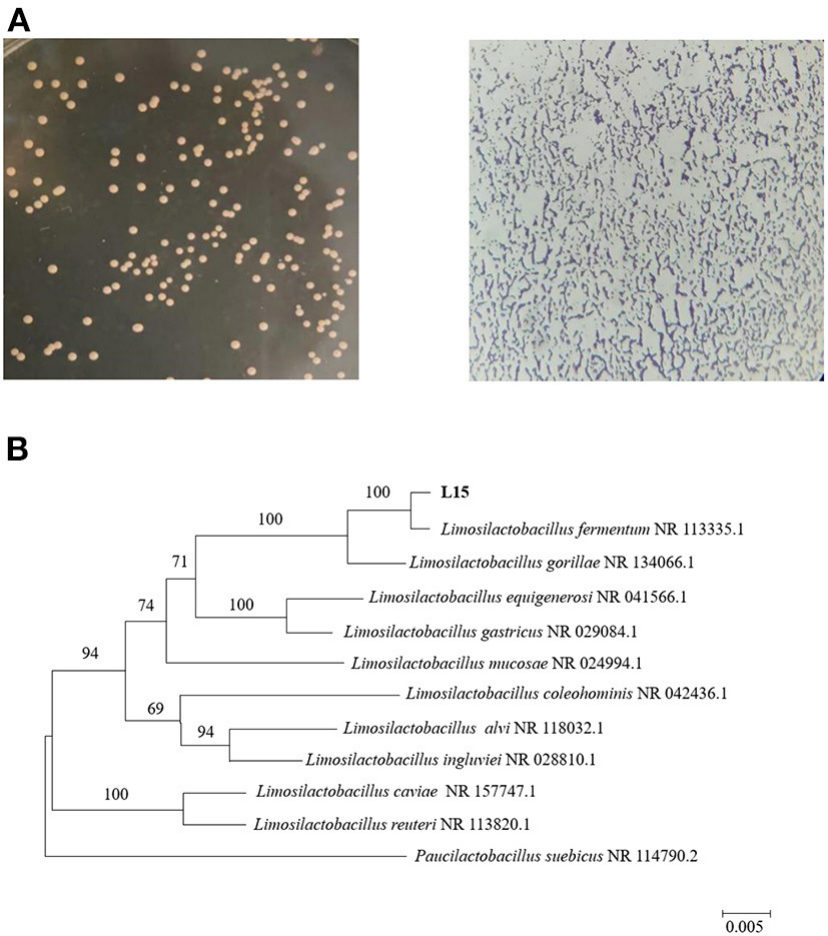
Identification of strain L15. **(A)** Morphology of screened strain L15 on MRS plate and under the microscope. **(B)** Phylogenetic tree based on 16S rRNA gene sequences of strain L15. The evolutionary history was inferred using the Neighbor-Joining method, scale bar represents 0.01 nucleotide substitution per position.

The 16S rRNA gene sequence was submitted to the National Center of Biotechnology Information with accession number OP616039. 16S rRNA-directed phylogenetic analysis primarily supported the delimitation of strain L15 as *Limosilactobacillus fermentum* (formerly named *Lactobacillus fermentum*) (Figure 2). *L. fermentum* has been identified as one of the probiotics that can be used in food, indicating that the L15 strain can be safely used as a dietary supplement.

### Preparation and optimization of corn fermented milk

Maize proteins and their derived peptides have become an essential source of bioactive peptides (40). The protein content is increased to 60% in the crushed corn. In addition to physical fragmentation, enzymatic, chemical hydrolysis, and biofermentation are also used to produce protein hydrolysates (41). In order to improve the antioxidant capacity of corn fermentation also, in this paper, corn was fermented with *Bacillus subtilis*, which was enzymatically digested by alkaline protease and they were fermented into fermented milk. Optimal conditions for corn enzymatic digestion were obtained by single-factor experiments with response surface optimization (Supplementary Table S1). The optimal enzymatic solution for corn (OEC) was determined as follows: 9.82% maize addition, 6.53% alkaline protease addition, pH = 9.19, and temperature 49.53°C (Supplementary Figure S1). The optimal fermentation protocol for *Bacillus subtilis* (OFB) was 6% inoculum, 18 h fermentation time, pH = 8, 37°C fermentation temperature, and TEAC of the ferment solution was 0.748 ± 0.016 mM (Supplementary Figure S2). The orthogonal results are shown in Supplementary Table S2. The optimal conditions for fermented milk were 9% (v/v) for OFB addition, 3% (v/v) for the first fermenting agent (*L. fermentum* L15), 2% (v/v) for the second fermenting agent (direct injection fermenting agent), and 8% (w/v) for sucrose addition. The peptide content and TAEC of fermented milk were 0.914 ± 0.009 mg/mL and 0.781 ± 0.020 mmol/L, respectively (Supplementary Figures S3A–D). The pH and viable cell count of corn fermented milk during fermentation were determined. As shown in Supplementary Figure S3E, following 6 h of fermentation, the pH of the fermented milk dropped to below 4.5. The rate of viable bacteria in the fermented milk was increasing, and the content of viable bacteria reached about 10 log_10_ CFU, and the water-holding capacity of the fermented milk was 57.48 ± 0.01%, all of which were in accordance with the reported requirements of the marketed fermented milk (42).

### Purification of antioxidant peptides

**Figure 3:**
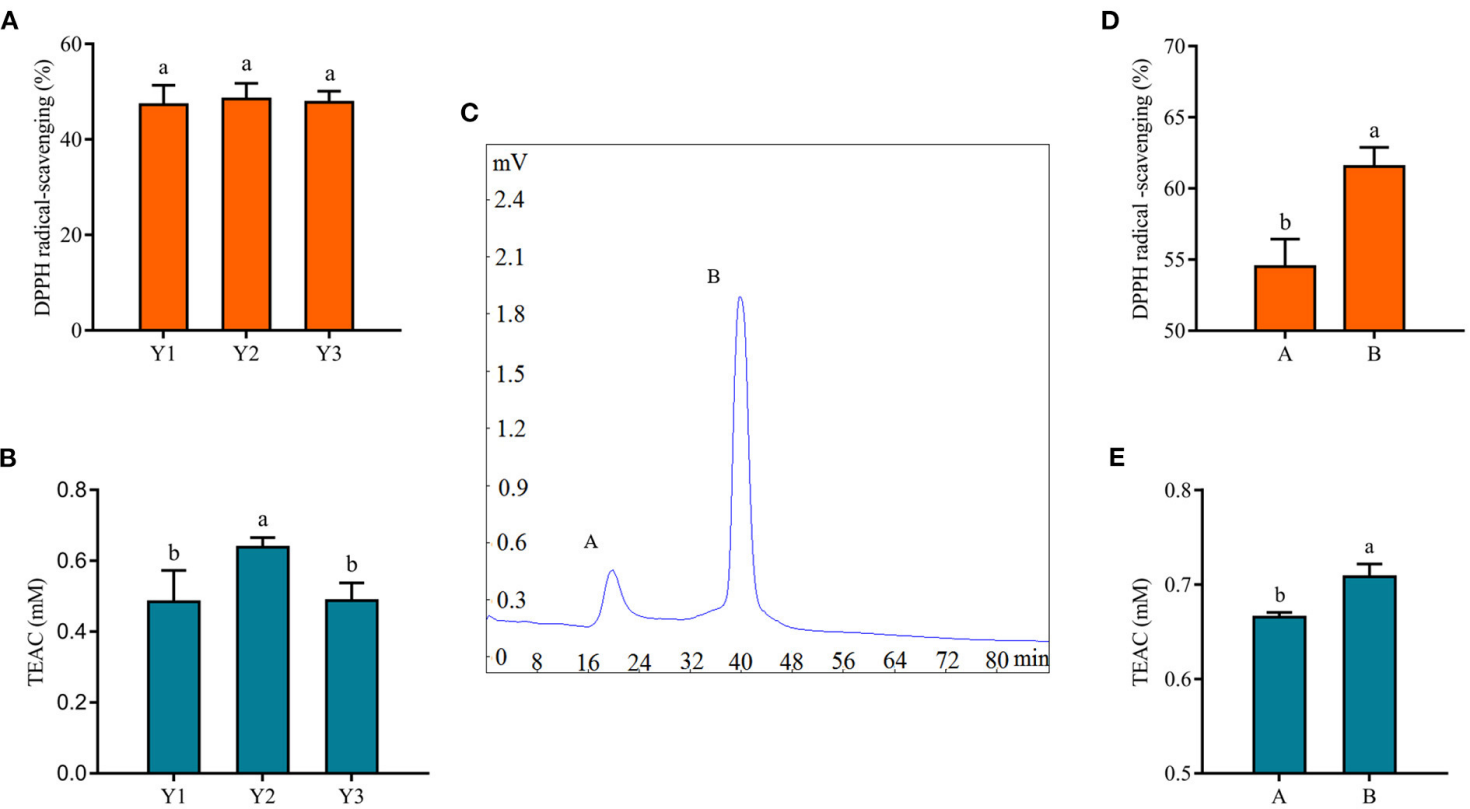
Isolation of antioxidant peptides from fermented yogurt. The DPPH radical scavenging ability **(A)** and TEAC **(B)** of each fraction are separated by centrifugal ultrafiltration. **(C)** Gel chromatogram of component Y2. The DPPH radical scavenging ability **(D)** and TEAC **(E)** of component Y2 are separated by centrifugal ultrafiltration. Different lowercase letters indicate significant differences between groups (*p* < 0.05).

The antioxidant activity of protein hydrolysates and peptides was related to their molecular weight (18). Antioxidant peptides in fermented milk were purified by centrifugal ultrafiltration and gel chromatography separation in this study. After centrifugal ultrafiltration, three fractions, named Y1, Y2, and Y3, were separated according to their molecular weights of 3 and 10 kDa, and their antioxidant properties were evaluated. Figures 3A,B showed the DPPH radical scavenging rate and TEAC content of these fractions. There was no significant difference in the DPPH radical scavenging rate of three fractions in Figure 3A (*p* > 0.05), while Y2 had a stronger antioxidant capacity in Figure 3B (*p* < 0.05), which might be related to the fact that the DPPH radical scavenging rate method was more suitable for alcohol-soluble substances (43). The supernatant of the fermented milk sample was used for subsequent analysis, and the results of the DPPH tests were not as significant as TEAC after centrifugation.

Subsequently, Y2 was separated by gel chromatography Sephadex G-25, and we collected fractions A and B in Figure 3C to evaluate their antioxidant capacity. Among them, the shortest retention time of component A indicated the largest molecular weight, while the longest retention time of B indicated the smallest molecular weight. Figures 3D,E clearly showed that the antioxidant capacity of component B was stronger. Similarly, Hernández-Ledesma et al. (44) isolated 10 different peptide fractions from commercial fermented milk by reversed-phase high-performance liquid chromatography and showed that one of the fractions with significant antioxidant activity consisted of antioxidant peptides. In addition, it was shown that the antioxidant activity of hydrolysates was closely related to their molecular weight distribution, and peptides or protein hydrolysates with small molecular weight could react better and more fully with free radicals during the oxidation process (45), which was consistent with the results of this study.

### Sequence and activities of the antioxidant peptides

**Figure 4:**
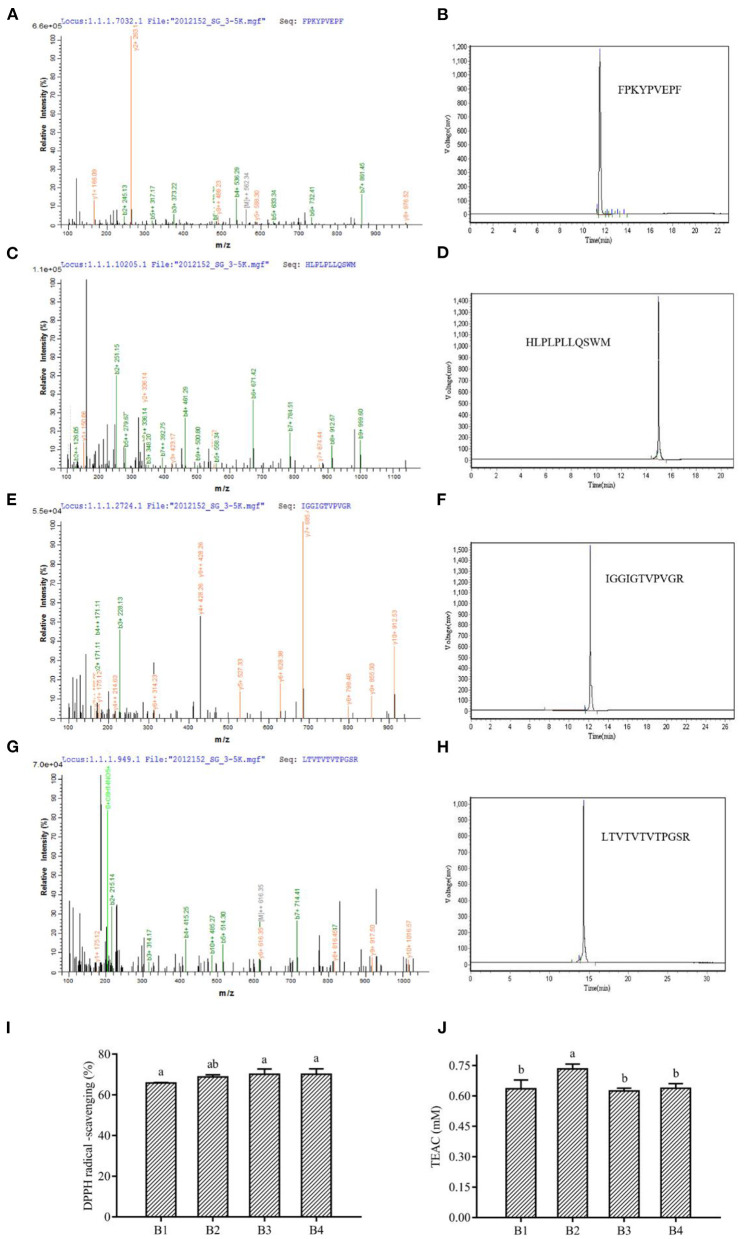
Mass spectrum and the primary structure of the peptide **(A)** FPKYPVEPF, **(C)** HLPLPLLQSWM, **(E)** IGGIGTVPVGR, and **(G)** LTVTVTVTPGSR. HPLC of synthetic peptides FPKYPVEPF **(B)**, HLPLPLLQSWM **(D)**, IGGIGTVPVGR **(F)**, and LTVTVTVTPGSR **(H)**. The DPPH radical scavenging ability **(I)** and TEAC **(J)** of peptides. B1, peptide FPKYPVEPF. B2, peptide HLPLPLLQSWM. B3, peptide IGGIGTVPVGR. B4, peptide LTVTVTVTPGSR. Different lowercase letters indicate significant differences between groups (*p* < 0.05).

The sequence identification of the isolated and purified fraction B was performed by LC-MS/MS. Due to the complex protein composition in corn fermented milk, a large number of peptide sequences were observed in this fraction. The antioxidant peptide capacity is related to the antioxidant peptide length. Lower molecular weight peptides had been reported to have higher antioxidant capacity. This is because lower molecular weight can readily react with lipid radicals, thus reducing free radical-mediated lipid peroxidation (35). We selected peptides with no more than 15 amino acids for further screening. Most of the peptides were derived from casein, glycoprotein, and lactoglobulin in milk, and a small percentage was derived from maize. We selected two peptides synthesized from maize and selected antioxidant peptides of bovine milk origin that have been reported as positive controls (Supplementary Table S3). The antioxidant peptide activity was related to the composition and sequence of amino acids, which had a significant effect on the antioxidant activity of the peptide (35). The peptides containing His, Leu, Tyr, Met, Pro, Trp, Phe, and Val were reported to have stronger antioxidant activity (46). Four peptides showed the presence of these amino acids in our study, namely FPKYPVEPF, HLPLPLLQSWM, IGGIGTVPVGR, and LTTVTVTPGSR, named B1, B2, B3, and B4. Therefore, the four peptides were resynthesized and their antioxidant activities were verified, and it was found that all four sequences of the peptides had more than 60% DPPH radical scavenging rate and more than 0.6 mmol/L TEAC content (Figure 4). Analysis of peptide data from the BIOPEP database showed that FPKYPVEPF and HLPLPLLQSWM were derived from bovine milk proteins. Among them, peptides FPKYPVEPF (47), and HLPLPLLQSWM (48) had been identified as antioxidant peptides by different researchers. Antioxidant peptides isolated from soybean hydrolysate and egg white hydrolysate have also been reported to be attributed to histidine due to the protons providing the ability of the imidazole moiety (49). It has also been reported that the antioxidant activity of peptides in β-casein trypsin hydrolysates VKEAMAPK is derived from methionine (50). Methionine residues in β-casein are preferred targets for oxidation, and these may be due to the release of -SH groups contributing to the antioxidant properties (51). Aromatic amino acids, such as Tyr and Phe, enhance antioxidant activity by effectively scavenging free radicals by providing protons like electron-deficient free radicals (52). Moreover, it has been demonstrated in our previous studies that bioactive peptide sequences containing two proline and one valine contribute to antioxidant activity (8).

The antioxidant capacity of IGGIGTVPVGR was predicted but not verified by Lu et al. (53) through the characterization and quantification of functional proteins and bioactive peptides produced during the enzymatic digestion of *Hermetia illucens* larvae fed with food wastes. Similarly, Karami et al. (54) isolated IGGIGTVPVGR peptide by proteinase K digestion of wheat germ and demonstrated its ability to inhibit angiotensin-converting enzyme (ACE). Interestingly, the present study found that IGGIGTVPVGR derived from maize proteins showed better antioxidant capacity, indicating adequate hydrolysis of maize by L. fermentum and alkaline proteases (serine protease) (Figures 4I,J). Besides, the peptide LTTVTVTVTPGSR from *L. fermentum* L15 fermented corn cow's milk protein hydrolysate, derived from corn protein, had strong antioxidant activity, which was consistent with previous studies that the free radical scavenging ability of proteins or peptides was related to serine content (55). Similarly, n-termini containing hydrophobic amino acids, including valine or leucine, had been reported as strong oxidants (56). Hydrophobic amino acid residues such as valine or leucine also contribute to the formation of antioxidant peptides.

At the same time, alkaline proteases could digest proteins from maize into peptides with antioxidant activity. Based on the digestion site, we found that the formation of IGGIGTVPVGR, and LTTVTVTPGSR matched perfectly with Arg (R), the digestion site of a serine protease (alkaline protease). In addition, the duplication of 2–3 amino acid residues in the peptide might be related to its antioxidant activity (57). Interestingly, our study revealed that the peptide IGGIGTVPVGR had successive repeating hydrophobic amino acid residues and in our identification results, 1 mg/mL of IGGIGTVPVGR showed a scavenging rate of 70.07 ± 2.71% of DPPH radicals and a total antioxidant capacity (ABTS method) measured at 0.62 ± 0.01 mmol/L (Figures 4I,J).

**Figure 5:**
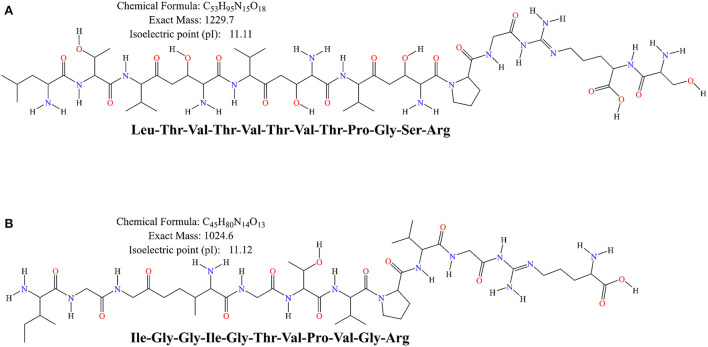
The primary structure of the peptides LTVTVTVTPGSR **(A)** and IGGIGTVPVGR **(B)** was generated by ChemBioDraw Ultra (13.0) software.

Moreover, the difference in antioxidant activity of the experimental samples could be attributed to the ability of the cultures to produce antioxidant peptides during the fermentation process, which contributes to the antioxidant activity. Virtanen et al. (58) found that the antioxidant activity was dependent on the strain used and increased during the fermentation process. Ferments of *L. casei* enhanced the antioxidant activity of cheddar cheese during ripening (59). It has also been observed that the antioxidant activity of fermented milk was strain dependent, and that fermented milk could be used as a carrier of antioxidant probiotic LAB from non-dairy sources (60). Farvin et al. (61) found that the high antioxidant activity of fermented milk may be due to the release of antioxidant peptides by lactic acid bacteria during fermentation, which is consistent with our results. LAB has a rich enzyme system that can effectively break down proteins. Both corn and bovine milk are rich in proteins, and this study found that the antioxidant peptides from bovine milk protein and those from corn protein both have superior antioxidant capacity. Except for IGGIGTVPVGR, LTTVTVTPGSR showed a scavenging rate of 70.07 ± 2.77% for DPPH radicals and total antioxidant capacity (ABTS method) was determined to be 0.64 ± 0.02 mmol/L (Figures 4I,J). The primary structures of corn-derived peptides IGGIGTVPVGR and LTTVTPGSR isolated from corn fermented milk were shown in Figure 5.

## Conclusion

Overall, *L. fermentum* L15, a high antioxidant peptide-producing bacteria, was screened to ferment corn enzymatic digest and milk to produce an artificial corn fermented milk. Two novel maize-derived antioxidant peptides, IGGIGTVPVGR and LTTVTVTPGSR, were extracted from fermented milk by ultrafiltration purification and LC-MS/MS and were validated for their activity. This research has the potential to be applied to the development of healthy antioxidant products from natural foods, and it provides a theoretical basis for functional food development and also offers a new practical basis for exploring bioactive food-derived peptides.

## Data availability statement

The original contributions presented in the study are publicly available. The data presented in the study are deposited in the NCBI repository, accession number OP616039. This data can be found here: https://www.ncbi.nlm.nih.gov/nuccore/OP616039.1/.

## Author contributions

JX: writing—original draft, data curation, investigation, methodology, and formal analysis. YC: conceptualization, writing—review and editing, data curation, formal analysis, investigation, and project administration. DP: conceptualization, project administration, funding acquisition, validation, and supervision. XF: data curation, methodology, and supervision. ZS: formal analysis, visualization, resources, and methodology. ML: resources and methodology. XZ: conceptualization and project administration. ZW: conceptualization and formal analysis. All authors contributed to the article and approved the submitted version.

## Funding

This work was financially supported by Natural Science Funding of China (31972048), the National Key R&D Program of China (2021YFD2100104), and the National Natural Science Foundation of China (32272339).

## Conflict of interest

The authors declare that the research was conducted in the absence of any commercial or financial relationships that could be construed as a potential conflict of interest.

## Publisher's note

All claims expressed in this article are solely those of the authors and do not necessarily represent those of their affiliated organizations, or those of the publisher, the editors and the reviewers. Any product that may be evaluated in this article, or claim that may be made by its manufacturer, is not guaranteed or endorsed by the publisher.
