# Supplementary material for: Isolation, identification, and characterization of corn-derived antioxidant peptides from corn fermented milk by Limosilactobacillus fermentum
Source: Front Nutr. 2022 Nov 9;9:1041655. doi: 10.3389/fnut.2022.1041655 (PMC9681995; doi:10.3389/fnut.2022.1041655)
Supplement: Supplementary file 1 [file Data_Sheet_1.docx]

Supplementary Material

# Supplementary Figures and Tables

## Supplementary Tables

**Table S1.** ANOVA of the response variables for the DH.

**Table S2.** Orthogonal optimization results for TEAC.

**Table S3.** Screening of target peptides from corn fermented milk.

# Table S1

Table S1 ANOVA of the response variables for the DH

| Source | DF | MS | F | P |
| --- | --- | --- | --- | --- |
| Model | 14 | 44.73 | 156.84 | < 0.0001** |
| A | 1 | 21.09 | 73.96 | < 0.0001** |
| B | 1 | 26.08 | 91.43 | < 0.0001** |
| C | 1 | 6.63 | 23.25 | 0.0003** |
| D | 1 | 11.21 | 39.31 | < 0.0001** |
| AB | 1 | 2.1 | 7.37 | 0.0168* |
| AC | 1 | 0.83 | 2.9 | 0.1105 |
| AD | 1 | 17.94 | 62.88 | < 0.0001** |
| BC | 1 | 19.62 | 68.8 | < 0.0001** |
| BD | 1 | 18.88 | 66.19 | < 0.0001** |
| CD | 1 | 49.28 | 172.78 | < 0.0001** |
| A^2^ | 1 | 226.45 | 793.94 | < 0.0001** |
| B^2^ | 1 | 25.72 | 90.16 | < 0.0001** |
| C^2^ | 1 | 217.25 | 761.69 | < 0.0001** |
| D^2^ | 1 | 177.92 | 623.79 | < 0.0001** |
| Residual | 14 | 0.29 |  |  |
| Lack of fit | 10 | 0.35 | 2.56 | 0.1894 |
| Pure error | 4 | 0.14 |  |  |
| Cor total | 28 |  |  |  |

***P* < 0.01, extremely significant; **P* < 0.05, significant. DF refers to degrees of freedom, MS refers to mean square, F and P refer to F and p-values, respectively.

# Table S2

Table S2 Orthogonal optimization results for TEAC

| Source | A | B | C | D | TEAC (mM) |
| --- | --- | --- | --- | --- | --- |
| 1 | 1 | 1 | 1 | 1 | 0.691±0.013 |
| 2 | 1 | 2 | 2 | 2 | 0.730±0.023 |
| 3 | 1 | 3 | 3 | 3 | 0.693±0.012 |
| 4 | 2 | 1 | 3 | 3 | 0.696±0.016 |
| 5 | 2 | 2 | 2 | 1 | 0.692±0.018 |
| 1 | 2 | 3 | 1 | 2 | 0.695±0.021 |
| 7 | 3 | 1 | 3 | 2 | 0.734±0.011 |
| 8 | 3 | 2 | 1 | 3 | 0.690±0.018 |
| 9 | 3 | 3 | 2 | 1 | 0.728±0.026 |
| K1 | 2.113 | 2.121 | 2.075 | 2.111 |  |
| K2 | 2.084 | 2.112 | 2.151 | 2.159 |  |
| K3 | 2.152 | 2.116 | 2.151 | 2.078 |  |
| k1 | 0.704 | 0.707 | 0.692 | 0.704 |  |
| k2 | 0.695 | 0.704 | 0.717 | 0.720 |  |
| k3 | 0.717 | 0.705 | 0.708 | 0.693 |  |
| R | 0.023 | 0.003 | 0.025 | 0.027 |  |

A, B, C and D refer to the amount of inoculum, time, pH and temperature, respectively.

**Table S3**

| Peptide sequence | MH+ | m/z | RT (min) | Length | Isoelectric point (pI) | Hydrophobicity | Protein accession |
| --- | --- | --- | --- | --- | --- | --- | --- |
| PKYPVEPF | 1122.574 | 562.2943 | 27.4667 | 9 | 6.61 | +10.16 Kcal * mol^-1^ | A0A452DHW7/BOVIN |
| HLPLPLLQSWM | 1333.718 | 667.8681 | 34.9333 | 11 | 7.69 | +3.98 Kcal * mol ^-1^ | A0A452DHW7/BOVIN |
| IGGIGTVPVGR | 1024.602 | 513.3087 | 16.65 | 11 | 11.12 | +11.54 Kcal * mol ^-1^ | EF1A/MAIZE |
| LTVTVTVTPGSR | 1229.696 | 615.8563 | 12.1167 | 12 | 11.11 | +9.83 Kcal * mol ^-1^ | K7V965/MAIZE |

MH+ refers to the molecular mass and m/z to the mass-to-charge ratio.

## Supplementary Figures


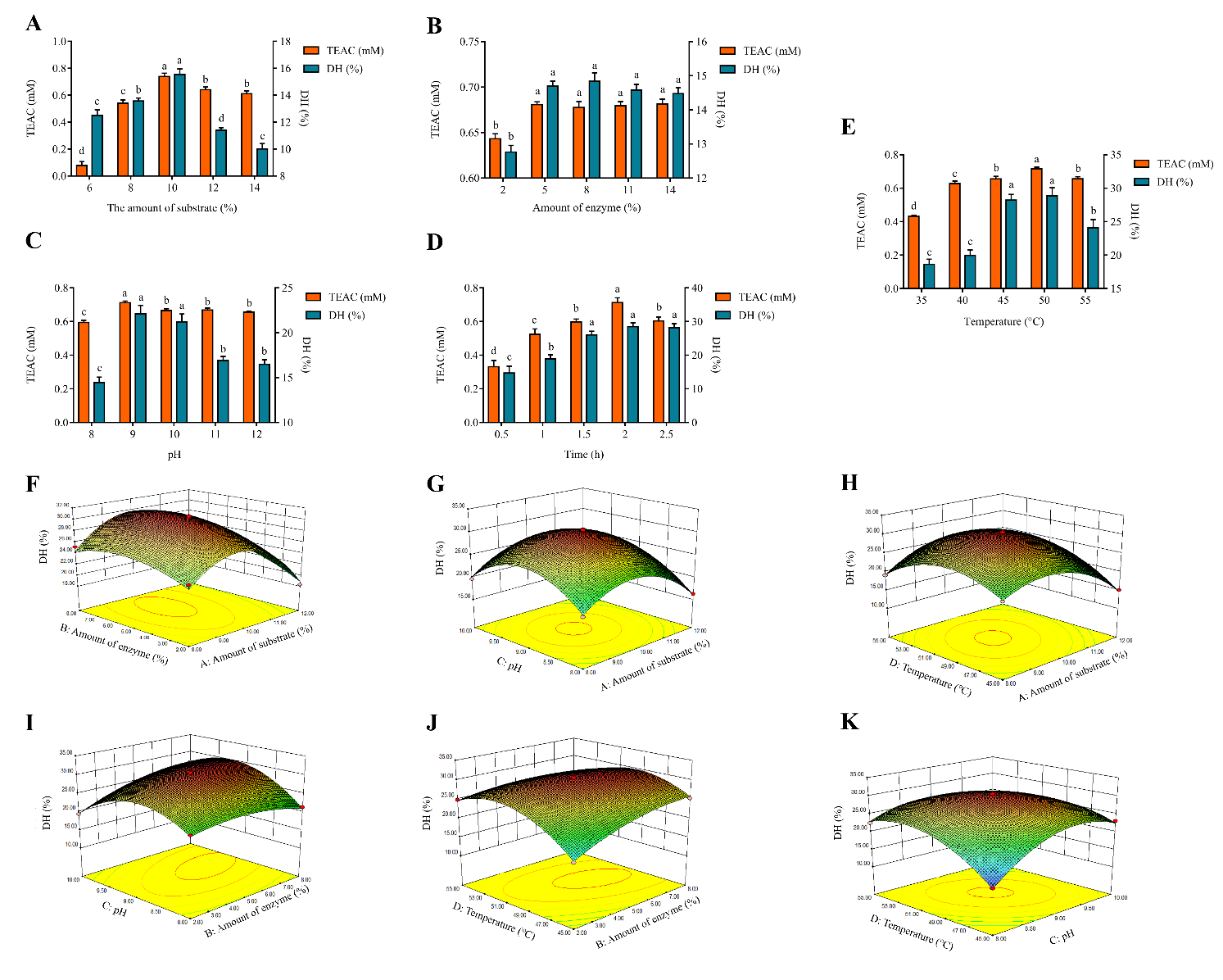


**Figure S1.** A-E are the conditions of the optimal enzymatic digestion solution for corn. The effects of substrate addition **(A)**, enzyme **(B)**, pH **(C)**, enzymatic digestion time **(D)** and temperature **(E)** on the degree of hydrolysis (DH) and TEAC of corn. F-K are response surface plots of the effect of variables on DH. **(F)** amount of enzyme and amount of substrate; **(G)** pH and amount of substrate; **(H)** temperature and amount of substrate; **(I)** pH and amount of enzyme; **(J)** temperature and amount of enzyme; **(K)** temperature and pH Different letters indicate significant differences between groups (*P* < 0.05).

**
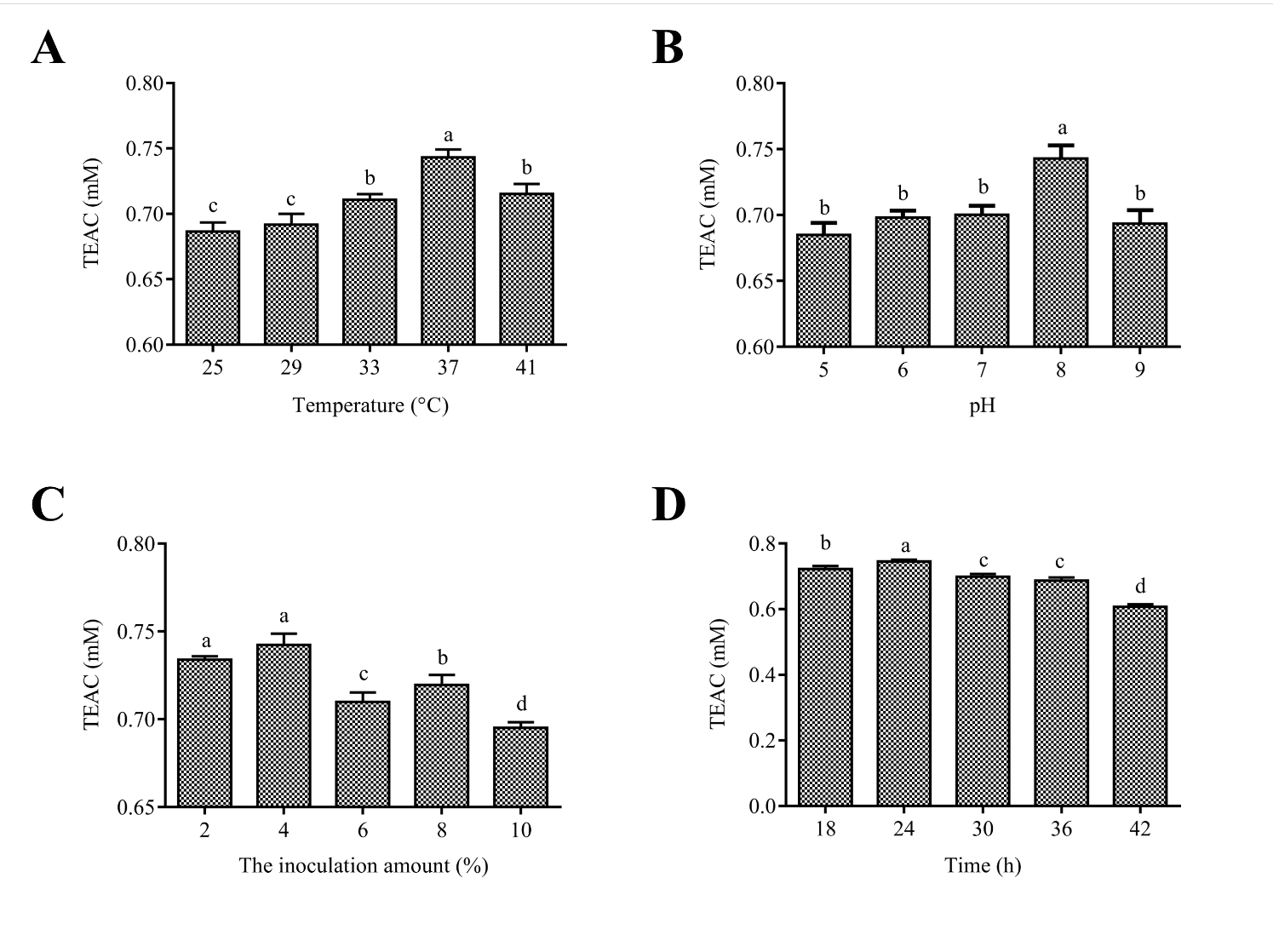
**

**Figure S2.** Optimal fermentation conditions for Bacillus subtilis (OFB). Effects of temperature **(A)**, pH **(B)**, inoculation amount **(C)** and time **(D)** on TEAC of corn fermentation broth. Different letters indicate significant differences between groups (*P* < 0.05).


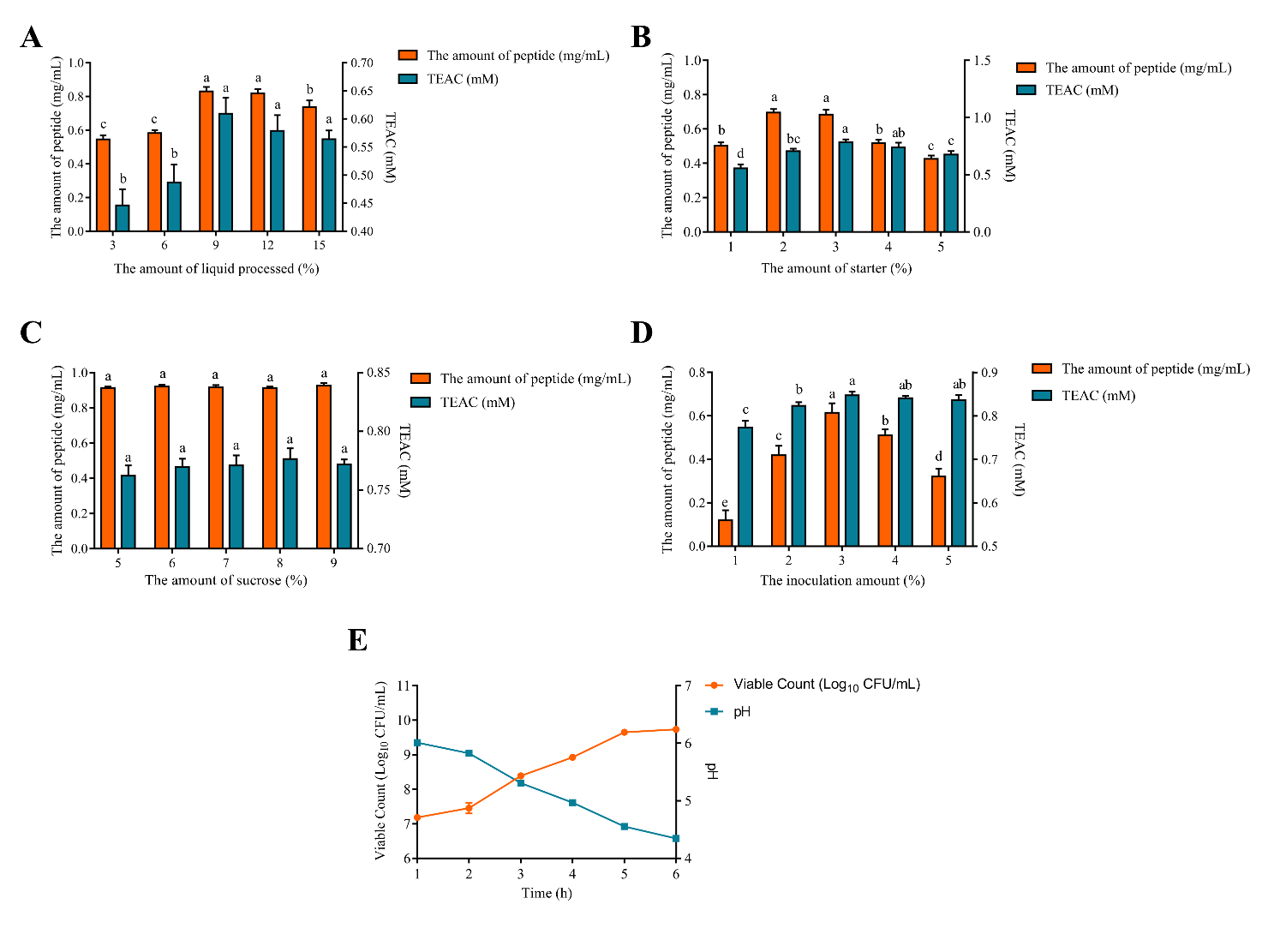


**Figure S3.** Optimal fermentation process of yogurt. Effects of OFB addition **(A)** starter addition **(B)**, sucrose addition **(C)** and inoculation of *L. fermentum* L15 **(D)** on peptide content and TEAC of yogurt production. **(E)** pH and live cell count during yogurt fermentation. Different letters indicate significant differences between groups (*P* < 0.05).
